# Sex Differences in Dopamine Receptors and Relevance to Neuropsychiatric Disorders

**DOI:** 10.3390/brainsci11091199

**Published:** 2021-09-11

**Authors:** Olivia O. F. Williams, Madeleine Coppolino, Susan R. George, Melissa L. Perreault

**Affiliations:** 1 Department of Biomedical Sciences, Collaborative Program in Neuroscience, University of Guelph, Guelph, ON N1G 2W1, Canada; williamo@uoguelph.ca (O.O.F.W.); coppolim@uoguelph.ca (M.C.); 2 Department of Pharmacology, University of Toronto, Toronto, ON M5S 1A4, Canada; s.george@utoronto.ca; 3 Department of Medicine, University of Toronto, Toronto, ON M5S 1A4, Canada

**Keywords:** dopamine receptors, sex differences, heteromers, neuropsychiatric disorders

## Abstract

Dopamine is an important neurotransmitter that plays a key role in neuropsychiatric illness. Sex differences in dopaminergic signaling have been acknowledged for decades and have been linked to sex-specific heterogeneity in both dopamine-related behaviours as well as in various neuropsychiatric disorders. However, the overall number of studies that have evaluated sex differences in dopamine signaling, both in health and in these disorders, is low. This review will bring together what is known regarding sex differences in innate dopamine receptor expression and function, as well as highlight the known sex-specific roles of dopamine in addiction, depression, anxiety, schizophrenia, and attention deficit hyperactivity disorder. Due to differences in prognosis, diagnosis, and symptomatology between male and female subjects in disorders that involve dopamine signaling, or in responses that utilize pharmacological interventions that target dopamine receptors, understanding the fundamental sex differences in dopamine receptors is of vital importance for the personalization of therapeutic treatment strategies.

## 1. Introduction

Dopaminergic signaling is fundamental to a number of neurobiological processes that are required for cognition [1,2,3], emotion [4], movement [5], and reward [3,6,7,8]. Dopamine signaling is mediated through G protein coupled receptors, D1, D2, D3, D4, and D5, that are categorized into two subfamilies, D1-like and D2-like, based on their structure and function [9,10,11,12]. D1-like receptors (D1 and D5) are excitatory and coupled intracellularly to canonical stimulatory Gs/olf proteins, whereas D2-like receptors (D2, D3, and D4) are inhibitory and coupled to Gi/Go proteins [11,12]. Dopamine-induced biological responses are therefore dependent on numerous factors including the subtype of dopamine receptor expressed, the receptor density, cell type, and brain region in which the receptors are located [11,13,14,15]. The type of response is further complicated as dopamine receptors can exist not only as homomeric complexes, but as heteromeric species that often exhibit pharmacological and cell signaling properties that are distinct from its constituent receptors [7,16,17,18,19,20,21].

An abundance of information exists on the role of the dopamine system in the brain. However, little is known regarding fundamental sex differences in dopamine function, despite decades old evidence from human [22,23] and animal [24,25,26,27] studies showing that sexual dimorphisms in dopamine receptor expression and function exist innately, as well as in response to various stimuli. In the years since, some additional clinical and preclinical evidence has emerged supporting the notion that the dopamine system of men and women is functionally distinct. A significant proportion of this evidence has come from studies of neuropsychiatric disorders, such as addiction or depression, disorders involving dopamine that often display sex differences in prevalence, symptomatology, and treatment responsiveness [28,29,30,31,32,33,34]. For example, clinical imaging studies have revealed brain region- and sex-specific variations in dopamine transporter (DAT) availability in depression [35], and dopamine D2-like receptor densities with nicotine addiction [36,37]. Animal studies have also been particularly useful at providing some understanding as to how the dopamine system may differ innately between males and females, with reports evaluating sex differences in dopamine release [38,39], and dopamine receptor expression, in both adult animals and during development [15,40,41,42,43]. As well, differences in functional and behavioural responses to dopamine receptor agonists and antagonists have been observed, which include sex-specific differences in decision making and learning [44,45], anxiety and depression-like behaviour [43,46], and reward [39,47,48].

Overall, our lack of knowledge surrounding sex differences in dopamine function in health and disease stems from the fact that the majority of past clinical and preclinical studies have focused predominantly on male subjects. Or, in those studies that have used both male and female subjects, sex and gender has not been considered as an experimental variable. This has resulted in a significant knowledge gap that impacts sex- and gender-specific determinants of health, and the understanding and treating of disorders involving this neurotransmitter in males and females. For instance, there have been many neuropsychiatric disorders in which dopamine has been shown to play a key neuropathological role, disorders that include not only the aforementioned depression [49,50] and addiction [51], but also schizophrenia [52], bipolar disorder [53,54], obsessive compulsive disorder [55], attention deficit hyperactivity disorder [56], and autism spectrum disorder [57]. If novel and more tailored treatment strategies are to be developed, then inclusivity of both sexes in research examining disorder neuropathology, as well as treatment responses, is a crucial step forward. The focus of this review is to therefore disseminate what is presently known regarding sex differences in innate dopamine receptor expression and function, incorporating both clinical studies and what has been learned from preclinical research. Sex differences in dopamine function in various neuropsychiatric disorders, such as addiction, depression/anxiety, schizophrenia, and attention deficit hyperactivity disorder (ADHD), will also be highlighted.

## 2. Sex Differences in D1-Like Receptors

The D1-like class of dopamine receptors (D1 and D5) are stimulatory G protein-coupled receptors (GPCRs) that, when activated, couple to Gs/olf proteins to activate adenylate cyclase and promote the production of cyclic adenosine monophosphate (cAMP) [11,58,59]. There is high structural homology between the D1 and D5 receptor, which has made elucidating the discrete in vivo functional effects of the D1 and D5 receptor difficult, owing to a lack of subtype-specific pharmacological agonists and antagonists. In line with this, examining subtype-specific receptor expression by pharmacological or immunologic means has been historically problematic, although mRNA expression studies have played a critical role in delineating D1 or D5 receptor distribution. It is known that the D5 receptor has a higher affinity for dopamine than the D1 receptor, which shows greater constitutive activation in the absence of an agonist [60]. D1 and D5 receptors additionally have differing affinities for agonists and antagonists [61] and exhibit a widespread, but distinct, regional distribution in human, non-human primate and rodent brains [62,63,64,65].

D1 receptor mRNA shows very high expression in the striatum of non-human primates [66], a finding supported by more recent single cell RNA sequencing (scRNA-seq) studies both in non-human primates [67] and mice [67,68]. Indeed, the expression of striatal D1 receptor mRNA is localized to neurons with unique transcriptional profiles, profiles that vary depending upon the subregion in which the mRNA is expressed [67,68]. Dopamine D1 and D5 receptors both have high expression in cortical regions, although D5 receptor expression has been shown to be higher than the D1 receptor in the prefrontal cortex (PFC) of rodents [64]. D5 receptor-immunoreactivity has been shown in both interneurons, as well as in pyramidal neurons, which frequently co-express the D1 receptor [64]. However, in pyramidal neurons, there is only marginal anatomical overlap between the receptors, with the D5 receptor more commonly found in dendritic shafts and perikarya and little expression within dendritic spines [69]. As few papers have delineated the discrete functional effects of D1 and D5 receptors, for the purpose of this review, unless otherwise stated, the described findings will be inclusive of both D1 and D5 receptors (subsequently termed D1).

Overall, studies evaluating sex differences in D1 receptor expression are sparse, and those that do exist are preclinical, using rodent models. For example, in comparison to female rats, evidence indicates that male rats show a transient overproduction of dorsal striatal D1 receptors during puberty, a sex difference that does not persist into adulthood due to the subsequent pruning of the receptors [42]. In contrast, the overproduction of dopamine D1 receptors in the male rat nucleus accumbens (NAc) does persist into adulthood [42], with adult male rats showing significantly higher D1 receptor expression than that of their adult female counterparts [42,43]. Another study showed that 30-day-old juvenile female rats exhibited greater concentrations of D1 receptors in the cortex and the striatum compared to males [70]. Furthermore, rodent females appear to exhibit a greater D1:D2 receptor expression ratio in the infralimbic cortex throughout development compared to their male counterparts [41]. Yet, in the insular cortex, males exhibited a drastic increase in D1:D2 expression ratio throughout development [41]. Male rats also exhibit a more prominent increase in striatal D1 receptors early in development followed by a rapid decrease in dopamine D1 receptors in adulthood compared to females [40]. This increase in striatal D1 receptors in males during such a critical time is proposed to have a role in the expression of hyperactivity in attention deficit hyperactivity disorder (ADHD) [40], which is further explored in the ADHD section of this review. Environmental factors may also influence dopamine function, with preclinical research showing that early stress exposure induces sex-specific outcomes in D1 receptor mRNA expression and binding [71,72]. Specifically, early stress induced by maternal separation upregulated D1 receptor gene expression in the brain stem of male, but not female, rats [71]. In addition, prenatal alcohol exposure of rhesus monkeys increased D1 receptor binding in the PFC of male monkeys only [72].

Functional differences in dopamine D1 receptors also exist between males and females, with sex differences in responsivity to D1 receptor agonists reported [73,74]. For example, when exposed to a single systemic injection of D1 agonist SKF 81297 or SKF 82958, female rats exhibited an initial increase in locomotor activity, within the first 5 min, while this effect was not observed in males [74]. Although, both males and females exhibited an overall equal increase in agonist-induced locomotor responses across the entire testing period [74,75]. This agonist-induced increase in locomotion was attenuated in both sexes by the D1 antagonist SCH 23390, albeit with greater sensitivity in females [75]. Further, administration of a D1 receptor partial agonist, SKF 38393, infused directly into the NAc, reduced social interaction behaviour in female mice, with no effect in males [73]. Social learning was also impaired in both male and female mice when the D1 antagonist SCH 23390 was infused into the hippocampus, although males showed increased sensitivity to the drug [45,76]. Together, these findings indicate that sex differences exist in innate D1 receptor functional responses, which in turn influence behavioural outcomes.

## 3. Sex Differences in D2-Like Receptors

D2-like dopamine receptors (D2, D3, and D4) mediate inhibitory neurotransmission as they are coupled to Gi/Go proteins to reduce cAMP concentrations [77]. D2-like receptors demonstrate a greater affinity for dopamine compared to D1 receptors that supports differential roles for D1 and D2 receptor subgroups [12]. Of the dopamine D2-like receptors, the D2 receptor shows the greatest distribution and highest overall availability, particularly in cortical and subcortical regions [78,79,80,81], and therefore, has received greater attention in the literature compared to D3 and D4 receptors. As with striatal D1 receptors, D2 receptors also are localized to neurons that have a distinct subregion-dependent transcriptional profile [67,68]. Whereas D2-like receptors have been identified in the temporal cortex, frontal cortex, hippocampus, caudate, putamen, ventral striatum, and pallidum of humans [78], non-human primate and rodent studies have shown D3 receptors in the NAc, olfactory tubercle, dorsal subiculum, and amygdala in rodents [81], and D4 receptor localization in the cerebral cortex, hippocampus, substantia nigra, among other regions [82,83]. With D2-like receptors dispersed throughout various brain regions, they are involved in many critical functions and pathways, including memory and locomotion [84,85]. As with D1-like receptors, the majority of studies group dopamine D2-like receptors together (subsequently termed D2 receptors).

Similar to D1 receptors, studies examining sex differences in D2 receptor expression are somewhat limited. PET studies in human subjects have revealed that women have higher D2 receptor expression than men in the frontal and temporal cortices as well as the thalamus [23]. D2 receptor density in the striatum has also shown to decline with age in a sex-specific manner, with men experiencing a greater exponential decline compared to women [86]. Preclinical work in animals further supports sex differences in D2 receptor expression, with male rats expressing a higher density in the cortex, and female rats exhibiting higher density in the striatum [43,70]. Moreover, male rats expressed a greater increase in striatal D2 receptors throughout early development compared to females [40]. Aside from receptor densities, males demonstrate a higher basal activation of D2 receptors in the medial PFC compared to females, as measured by autoradiography using the D2/D3 receptor agonist quinpirole [87]. From a functional perspective, using the rat version of the Iowa gambling task, one study showed that administration of the D2 receptor antagonist eticlopride decreased advantageous responding in male, but not female rats, whereas administration of quinpirole decreased advantageous responding selectively in females [44]. Genetic knockout of the *Drd2* gene, selectively in neurons expressing the serotonergic transcription factor gene *Pet1*, also resulted in sex-specific alterations in behaviour in mice, with males showing increased sociability and females decreased acoustic startle responses [88].

With regard to the dopamine D3 receptor specifically, functional studies employing transgenic lines or selective pharmacological agents have been beneficial in delineating not only the functional importance of the D3 receptor, but in the identification of sex differences. In a transgenic reporter mouse model of dopamine D3 receptors, males and females were shown to differ in D3 receptor mRNA expression and its co-expression with either D1 or D2 receptor mRNA [81]. Specifically, in the NAc, male mice had greater co-expression of D3 and D1 receptor mRNA at both postnatal day 35 and 70, as well as greater co-expression of D3 and D2 receptor mRNA, whereas on postnatal day 35, females had a higher co-expression of D3 and D2 receptor mRNA compared to males [81]. When dopamine D3 receptor knockout (D3-/-) mice were evaluated, they exhibited hyperactivity [84]. However, female D3-/- mice showed higher activity in a running wheel compared to their male counterparts [84]. Further, both male and female D3-/- mice exhibited hyperalgesia, although females expressed significantly less nociceptive behaviours compared to their sex-matched wildtype litter mates [89]. Pharmacological studies have also been utilized, as the administration of the D2/D3 receptor agonist quinpirole to non-human primates elicited yawning in males to a greater degree compared to females [90]. It was established that yawning correlated with dopamine D3 receptor densities in various regions, including the globus pallidus, caudate nucleus, putamen, ventral pallidum, and hippocampus [90]. There is little information that exists on innate sex differences in dopamine D4 receptor expression and function; although, the antagonist clozapine, which has a high affinity for the D4 receptor, was found to increase hypothalamic D4 receptor expression to a greater extent in females compared to males [91].

## 4. Sex Differences in the Dopamine D1–D2 Receptor Heteromer

Although, traditionally, GPCRs, such as the dopamine receptors, have been previously depicted as monomeric entities, it is now widely accepted that GPCRs exist as oligomeric complexes [7,92,93,94]. In addition to homomeric receptor complexes, numerous reports have demonstrated that dopamine receptors can form heteromeric complexes with other subtypes of dopamine receptors as well as with other GPCRs or ion channels [7,16,17,18,95,96,97,98,99,100,101,102,103,104] that may exhibit discrete distributions in the brain with distinct pharmacological and functional properties from their constituent receptors. Co-expression of D1 and D2 receptor mRNA or protein has been previously shown in the PFC [105,106], striatum [68,106,107,108,109,110], and in various regions of the basal ganglia [108] of rodents. In the striatum, D1 and D2 receptors are predominantly segregated to discrete populations of medium spiny neurons, although it has been hypothesized that the subset of neurons that co-express both receptors may represent a third distinct neuronal pathway [14]. This idea is supported by a recent sc-RNA-seq study that showed striatal *Drd1a* and *Drd2* transcripts were co-expressed selectively in an MSN subtype that also expresses protocadherin 8 (Pcdh8) [68]. At a subregional level, striatal dopamine D1 and D2 receptors more commonly colocalize within the NAc, with highest co-expression in the shell subregion and lowest in the dorsal striatum [106,107,108,109,110].

A physical interaction between endogenously expressed striatal D1 and D2 receptors was first identified using quantitative confocal FRET in brain sections *in situ* [111,112,113]. Several studies have since demonstrated the formation of D1–D2 heteromers in the mesolimbic and basal ganglia circuitry of humans, non-human primates and rodents [106,108,109,114,115], although there has been some controversy as to the existence of the receptor complex under physiological conditions [116]. Unlike its constituent receptors, the D1–D2 heteromer couples to the Gq protein to increase intracellular calcium both in vitro in cells and neurons [43,104], as well as in vivo in the striatum [117], and to increase brain-derived neurotrophic factor (BDNF) expression and signaling [111,118]. Unfortunately, there is an almost total lack of research on sex differences in dopamine receptor heteromer expression and function, with only a single paper highlighting sex differences in dopamine D1–D2 receptor heteromer expression and function [43]. Hasbi et al. [43] demonstrated that in the caudate nucleus of non-human primate and in rat striatum, female animals expressed a higher density of D1–D2 heteromer complexes and a greater number of D1–D2 co-expressing neurons compared to males [43]. Interestingly, this sex difference in D1–D2 heteromer expression occurred despite the lower overall D1 receptor densities in females in these regions [43]. At a functional level, the sex difference in D1–D2 heteromer expression further led to corresponding differences in basal and heteromer-stimulated activities of two signaling pathways—BDNF/tropomyosin receptor kinase B (TrkB) and Akt/glycogen synthase kinase-3 (GSK-3)/β-catenin [43].

## 5. Sex Differences in Dopamine Receptors: Relevance to Neuropsychiatric Disorders

Dopamine receptors have long been known to play a key role in many neuropsychiatric disorders that include addiction [8,119], depression [49], anxiety [120], schizophrenia [52], as well as ADHD [121]. For all of these disorders, sex differences in prevalence, age of onset, and/or symptom presentation have been reported. Given the central role of dopamine, it is therefore likely that sex differences in dopaminergic tone are intimately involved in both sex-specific and disorder-specific characteristics. Added to this, evidence also indicates that there can be robust sex differences in pharmacological responses [122,123] that may have particular relevance to disorders such as addiction, as well as to pharmacological treatment strategies.

### 5.1. Addiction

Addiction commonly refers to a substance use disorder, in which an individual uses a specific substance, such as nicotine, alcohol or illicit drugs, to the extent of which they are no longer able to perform daily functions without resisting the urge to use such substances [124]. Men demonstrate a higher prevalence of substance use disorders than women [28,29,30]. However, women are more susceptible to harsher withdrawal symptoms and relapse compared to men [30,31,125]. Dopamine plays a central role in addiction that is mediated in large by the mesolimbic dopamine pathway, which sends neuronal projections from the ventral tegmental area (VTA) to the NAc [119,126,127], a pathway involved in regulating motivation, reinforcement and rewarding behaviours [128,129]. Disruptions in this system due to acute and chronic administration of addictive substances, or natural rewards, can lead to short- and long-term structural and functional alterations [128,129]. In addition to the VTA and NAc, other major brain regions such as the PFC, striatum, and the cingulate cortex are highly coupled to the reinforcing properties of substances causing addiction [36,130].

As outlined in the dopamine D1 receptor section of this review, males display a higher concentration of D1 receptors in the NAc compared to their female counterparts [42]. This may lead to a heightened susceptibility for males to develop addiction, as excitatory D1 receptors in the NAc are involved in the reinforcing properties of addictive substances [8]. Clinical imaging studies suggest that individuals with addiction display sex-specific differences in dopamine receptor expression and function, with most of the work centered around nicotine. For example, it has been demonstrated that men with moderate nicotine addiction express significantly lower D2 receptor availability in the striatum compared to women [36]. This lower D2 receptor availability in men was suggested to correlate to the greater vulnerability that exists for men to become nicotine dependent [36]. Furthermore, PET imaging studies have revealed that men and women with nicotine addiction differ in D2 receptor densities in the VTA, as women showed greater D2 receptor binding in this region [37]. Additionally, men who smoke cigarettes show a significantly greater activation within the ventral striatum compared to women, and as such, it was suggested that men may experience greater reinforcing effects of nicotine when they smoke [131]. Chronic nicotine administration was further shown to induce a sex-specific change in *Drd2* mRNA expression in rats, with females showing a greater nicotine-induced increase in *Drd2* gene expression in the striatum and PFC compared to males [132].

Preclinical work has also identified that repeated administration of nicotine induced sex-dependent alterations in D3 receptor expression measured by autoradiography [133]. Specifically, male rodents displayed a higher number of D3 receptors in the shell region of the NAc compared to females following nicotine exposure [133]. Moreover, sex-specific changes in dopamine levels as a result of nicotine withdrawal have been reported [134]. Female rodents demonstrated an increase in dopamine levels in the NAc during nicotine exposure, and a larger reduction in dopamine levels in the NAc during withdrawal compared to males [134]. Further, female rats that were neonatally exposed to quinpirole exhibited increased dopamine D2 receptor sensitivity [135]. When the effects of acute nicotine treatment during adolescence, in addition to neonatal quinpirole exposure, were examined, females expressed heightened locomotor activity, while males exhibited an increase in activity only after subchronic nicotine treatment during adolescence [135]. Cocaine administration (a potent DAT blocker) also shows sex-specific effects. Specifically, when the drug was administered to mice, there was an increase in D2-receptor-expressing neurons in the medial PFC and VTA that was greater in females compared to males [136]. Cocaine administration also reduced overall neuronal populations in these regions, which the researchers posited could be associated with the cognitive impairments associated with chronic cocaine use [136]. Overall, while it remains unproven whether men have a greater inherent susceptibility to addiction due to differences in baseline dopaminergic receptor expression, evidence suggests that this may be the case, while women appear to experience a greater change in dopaminergic activity as a result of addiction and withdrawal from addictive substances, such as nicotine.

### 5.2. Depression and Anxiety

Depression and anxiety disorders are amongst the most common illnesses worldwide, and though they can occur independently, they frequently co-occur with one another [137]. A widely replicated finding in epidemiology is that there is a higher incidence of depression in women than in men [32,33,34]. Although the degree of this difference depends on many factors including age, ethnicity, and socioeconomic status, and reporting bias may also be involved [138]. The involvement of dopamine in depression is well recognized, as reduced dopamine is believed to contribute to symptoms such as anhedonia, the lack of a sense of pleasure and reward [49]. Depression and anxiety studies have also shown the involvement of certain dopamine receptor gene polymorphisms [139], alterations in receptor binding [140] and region-specific expression [141] without accounting for sex.

Clinical work has shown that D1 receptor binding is altered in individuals with depression [142]. Specifically, compared to men, women with depression display a lack of an inter-hemisphere binding potential ratio for the D1 receptor within the dorsal putamen, which was observed in healthy women [142]. The women with depression exhibited a lower left hemisphere binding potential, which altered the inter-hemisphere binding potential ratio [142]. In regard to the dopamine transporter, men and women with depression exhibit an increase in striatal DAT availability [35]. However, as measured by single photon emission tomography scanning (SPECT), women with depression exhibited greater DAT binding than men, specifically within the caudate nucleus [35]. For both men and women with depression, DAT binding also showed region-specific normalization after treatment with the antidepressant bupropion, specifically in both the right and left caudate of women, and in the right caudate of men [35]. The significance of delineating right versus left caudate was to determine if laterality exists for DAT binding in depression in a sex-specific manner [35]; the researchers chose to do so based on previous findings suggesting stress responses could exhibit left or right regional bias for females and males, respectively [143]. Another study examined the effect of violent victimization and the relationship between depression-like symptoms and the dopamine D2 receptor gene (*DRD2*), while analyzing potential race- and sex-specific variations [144]. The researchers showed that violent victimization was associated with higher levels of depression symptoms in African American females when they carried at least one A1 allele of the *DRD2* gene, an effect not observed in Caucasian women or men of either race [144].

The importance of the dopamine D1–D2 receptor heteromer in the neuropathology of neuropsychiatric disorders such as addiction, schizophrenia, and depression has been identified [7,113,145,146]. However, sexual dimorphisms have only been reported in the context of depression-like behaviour and anxiety in rats [43]. Specifically, when male and female rats were administered the D1–D2 receptor heteromer agonist SKF 83959, females exhibited increased sensitivity to the pro-depressive and anxiogenic properties of the drug, effects that were abolished in both sexes by the pre-administration of an interfering peptide that blocks D1–D2 receptor heteromer function [43]. In addition, the anxiogenic effects of SKF 83959 were correlated to alterations in brain field potentials recorded from the NAc, specifically in the low frequency ranges [43]. Given that D1–D2 heteromer expression was shown to be higher in the NAc for females compared to male rats [43], and dopamine activity in the NAc is critically involved in depression [49], these findings suggest that D1–D2 heteromer function within the NAc may be involved in the increased female vulnerability to developing depression- and/or anxiety-like behaviour.

### 5.3. Schizophrenia

Schizophrenia is a complex neuropsychiatric disorder that affects both men and women with approximately equal incidence [147]. However, onset typically occurs earlier in men, in the late teens, whereas typical onset for women is in the early twenties [148,149]. Sex differences in symptomatology have also been reported, with females expressing more mood disturbances and positive symptoms and males displaying more negative symptoms [149]. Dopamine and its receptors are involved in the pathology of schizophrenia, as alterations in dopamine neurotransmission [150,151,152] and receptor densities [153] have been reported. For example, individuals with schizophrenia exhibit higher striatal D2 receptor density compared to neurotypical controls [153], a finding proposed to be indicative of the effective treatment response of antipsychotic drugs on positive symptoms such as delusions and/or hallucinations [124,150,153]. Conversely, D1 receptor hypofunction appears to play a significant role in the cognitive symptoms of schizophrenia [124,151].

Little is known about sex-specific effects in dopamine function in schizophrenia, despite the presence of sex differences in onset and symptomatology [149]. Of these studies, the focus is less on the neuropathology of schizophrenia, but rather on sex differences in antipsychotic treatment responses [38,47,91,154]. A clinical study analyzed findings from four distinct PET imaging studies: three on schizophrenia and one on bipolar disorder [154]. Sex differences in dopamine D2 receptor occupancy following administration of the atypical antipsychotic olanzapine was analyzed and it was shown that women required a lower dosage to achieve the same D2 receptor occupancy as men [154]. Preclinically, chronic administration of the atypical antipsychotic clozapine increased expression of D2 receptors in the hypothalamus of male rats, while it decreased D2 receptor expression in this region in females [91]. Further, this study showed that chronic administration of the typical antipsychotic haloperidol selectively increased expression of hypothalamic D2 receptors in male rats [91]. Another study that examined acute exposure to haloperidol in rats identified that haloperidol induced dopamine efflux in the striatum of both females and males, although to a much greater extent for the females [38]. Haloperidol has also been shown to have sex-specific behavioural effects in an operant responding task, with males exhibiting lower response to food than females with varying doses of the drug [47]. Therefore, although sex differences in dopamine receptor expression in schizophrenia have yet to be examined, the differential responses to haloperidol may indicate the presence of sex-specific differences in dopamine D2 receptor function.

### 5.4. Attention Deficit Hyperactivity Disorder

ADHD is a common neurodevelopmental disorder that is characterized by a persistent pattern of hyperactivity, impulsivity, and inattention during development [124]. The disorder is typically diagnosed during childhood, though it can also be diagnosed later in life, and it often persists over the individual’s lifespan [124,155]. There are sex differences in the symptomatic expression of the disorder, as females with ADHD exhibit less hyperactivity and externalizing behaviours compared to males with ADHD [156,157]. This difference in behavioural expression is proposed to result in diagnosis bias, where a greater proportion of males are diagnosed compared to females [156,157].

There are no clinical studies to date that have examined sexual dimorphisms in dopamine function in ADHD. However, preclinical work suggests that the differing dopamine receptor densities in the striatum, as exhibited in male and female rodents [40], may have relevance to the observed increased susceptibility of males to develop ADHD. As previously mentioned, male mice exhibited a more prominent increase in dopamine D1 and D2 receptors early on in development, and a more rapid loss of D1 receptors in adulthood, while females exhibited little overproduction and pruning of D1 and D2 receptors in development [40]. The initial rise in striatal D1 and D2 receptors in males aligned with the appearance of motor symptoms associated with an ADHD-like phenotype [40], suggesting that increased D2 receptors in the striatum may be linked to greater likelihood of the development of symptoms for males compared to females [40]. Furthermore, research on exposure to a common treatment for ADHD, methylphenidate (MPH), showed that the exposure of MPH to adolescent rats influenced striatal D2 receptor expression in adulthood in a sex-dependent manner [158]. Specifically, female rats exposed to MPH during adolescence showed increased D2 receptor expression in adulthood, whereas males under the same conditions did not [158]. Further, male rats that received MPH during adolescence performed worse on discrimination tasks during adulthood compared to males that did not receive MPH, an effect not observed in females [158]. It was suggested that this sex difference in learning abilities following adolescent MPH exposure may have long lasting impact on cognitive performance in adulthood [158].

## 6. Conclusions

Despite evidence demonstrating that there exist key innate sex differences in dopamine receptor expression and function within the brain, there has been a dearth of research that has focused on exploring sex differences in the dopamine system at both the clinical and preclinical levels. Indeed, with the majority of previous research focused solely on male subjects, this has led to a significant knowledge gap surrounding dopamine function in the female sex. Added to this, the lack of inclusion of sex as an important variable in research has greatly limited the advancement of knowledge surrounding neuropsychiatric disorders where dopamine plays a central role and in which there are established sex-based differences. The importance of sex in these disorders is only highlighted with the awareness that many of them demonstrate clear sex-specific differences in prevalence, symptom manifestation, and/or treatment responses. The delineation of clear-cut mechanistic adaptations that account for these differences would potentially assist in the development of more effective, mechanism-based, sex-specific therapies for these disorders. Thus, it is paramount that sex-dependent differences in dopaminergic activity in health and disease are identified.
